# Incidence and etiology of sudden cardiac arrest in Koreans: A cohort from the national health insurance service database

**DOI:** 10.1371/journal.pone.0242799

**Published:** 2020-11-25

**Authors:** Seung-Young Roh, Jong-Il Choi, Min Sun Kim, Eun Young Cho, Yun Gi Kim, Kwang-No Lee, Jaemin Shim, Jin Seok Kim, Young-Hoon Kim

**Affiliations:** 1 Division of Cardiology, Department of Internal Medicine, Korea University College of Medicine and Korea University Medical Center, Seoul, Republic of Korea; 2 Department of Biostatistics, Korea University College of Medicine, Seoul, Republic of Korea; 3 Division of Cardiology, Department of Internal Medicine, Ajou University Hospital, Suwon, Republic of Korea; King's College London, UNITED KINGDOM

## Abstract

The incidence of sudden cardiac arrest (SCA) in Asians is lower than that seen in Western populations, but there are few available data on the incidence and associated cardiac etiology of SCA in Asians. From 2002 to 2013, patients with SCA were analyzed using a cohort from the South Korean National Health Insurance Service (NHIS) coded database. Sudden unexplained death syndrome (SUDS) was defined as cryptogenic arrest, excluding that of non-cardiac origin, coronary artery disease (CAD), cardiomyopathy (CM), and valvular heart disease. During the 12-year study period, 5,973 patients (0.53%) from the total cohort of 1,125,691 had a cardiac arrest code. The overall incidence of arrest was 48.7 per 100,000 person-years (95% CI 16.6–18.0). The incidence of primary SCA excluding those of non-cardiac origin was 16.1 per 100,000 person-years (95% CI 15.4–16.8). It was higher in males than in females (18.1 vs. 14.1 per 100,000 person-years). CAD was the most common cause of SCA (59.4%), and followed by CM (13.9%). SUDS accounted for 14.7% of SCA events. The risk of SCA had increased gradually from over 25 years old. Heart failure, atrial fibrillation and hypertension are major factors associated with SCA incidence. Our findings outline epidemiologic data for SCA and the proportion of associated cardiac etiology leads SCA in a large population.

## Introduction

Cardiovascular disease is the leading cause of death worldwide, accounting for approximately 17 million deaths annually. Sudden cardiac arrest (SCA), which is defined as an unexpected, sudden death, accounts for approximately 25% of cardiovascular mortalities [1]. In the United States, the annual number of SCA events was estimated at 184,000–400,000 [2, 3]. The most frequent cause of SCA is coronary artery disease (CAD). Cardiomyopathy (CM), valvular heart disease (VHD), and infiltrative disease also are leading causes of SCA [4]. However, there are discrepancies between reports about the causes of SCA. In Western countries, CAD was the cause of at least 80% of all SCA cases [5]. However, Maruyama et al. showed that CAD caused only 25% of all SCA cases in Japan [6].

In autopsy data of sudden death victims, CAD is also a main cause, but sudden unexplained cryptogenic death accounts for 41.3% of SCA cases under 35 years of age [1]. “Sudden unexplained death syndrome” (SUDS) is defined as sudden arrest, non-cardiac etiologies have been excluded, and the heart has been found to be morphologically normal [7, 8]. In previous studies, SUDS accounted for 4.1% of SCA cases in the 16–64 age group [9]. Recent studies have shown that inherited cardiac arrhythmia (ICA) plays an important role in SUDS. Brugada syndrome, long or short QT syndrome, catecholaminergic polymorphic ventricular tachycardia, and early repolarization syndrome are well-known ICAs and often provoke SCA in patients with structurally normal hearts. SCA from these ICAs is likely to be attributed to SUDS because they are difficult to diagnose during post-mortem examination. Thus, the incidence of SCA and its specific cardiac etiology in the real world remain unknown.

The objectives of this study were to investigate the incidence and etiology of SCA in Korean using nationwide cohort data. We hypothesized that the incidence of SCA and the proportion of cardiac etiology are different in Korea compared to Western countries.

## Materials and methods

### Data source and study population

This study was conducted using the code database of the National Health Insurance Service National Sample Cohort (NHIS-NSC) in Korea. This database consists of 1,125,691 medical insurance subscribers who were selected from the NHIS database using stratified random sampling. The subscribers were followed for 12 years or until they were no longer considered eligible for health insurance due to death or emigration. During the follow-up period, the cohort was refreshed annually by adding a representative sample of newborns. The NHIS-NSC database includes demographic information, medical claims data, and disease diagnoses that were defined according to the International Classification of Diseases-10. The NHIS-NSC database includes demographic information, medical claims data, and disease diagnoses. The study protocol was approved by Institutional Review Board of Korea University Anam Hospital. Written informed consent was waived as the database maintains de-identified data and the anonymity of sampled individuals.

### Sudden cardiac arrest and sudden unexplained death syndrome

The study population consisted of subjects with cardiac arrest codes for cardiac arrest, ventricular fibrillation, sudden infant death, and sudden unexplained death (I46.0, I46.1, I46.9, I49.0, R95, R96, R96.0, R96.1). Arrest codes included both cardiac causes and cardiac arrests due to other organ failure. We excluded subjects with secondary cardiac arrest with a different critical primary cause, such as life-threatening cancer, cerebrovascular accident, trauma, infection, kidney failure, and lung disease (A00-09, A15-A19, A30-A49, A80-A89, B00-B09, B15-B19, B20-B24, B90-B94, C01-09, C11, C15-20, C22-26, C32, C34, C37, C40, C44, C45, C48-50, C52-56, C61, C63, C64, C66, C67, C70, C71, C73, C76, C80, C82-86, C90-92, C95, C97, D01, D16, D33, D35, D37, D42, D43, D46, D47, D64, D69, D73, D75, E85, E87, G04, G09, G11, G20, G30, G40, G61, G62, G71, G72, G80, G91, G93, I60-64, I69, I71, I85, I95, J09, J11, J15, J18, J42-46, J69, J84-86, K25-27, K55, K56, K65, K70, K74, K81, K83, K85, K90, L89, N17, N19, R54, R64, S00-79, T00-98).

The arrest cause for subjects was confirmed by analyzing the recorded main cause on the death certificate. The main death cause was filled in after evaluation during admission by a clinician or by autopsy. Among the survivors, the cause of arrest was confirmed by analyzing the disease, procedure, and surgery codes for the six months following the arrest. We estimated the prevalence of SUDS excluding CAD, VHD and dilated and hypertrophic CM. SCA by CAD was defined by myocardial infarction code and history of coronary intervention or surgical bypass at any time during the study period.

### Statistical analyses

Data for demographic and comorbidity variables are presented as numbers and percentages and compared using Chi-square tests. Continuous variables are described as means with standard deviations and were compared using Student’s t-test and analysis of variance (ANOVA). To examine what risk factor associated to SCA, we constructed Poisson regression models. The outcome in model was incidence of SCA. Covariates considered to be potential confounders were entered as fixed effects in the model and included age, sex, hypertension, diabetes, heart failure, chronic kidney disease, chronic lung disease, cerebrovascular accident, atrial fibrillation and malignancy. All p values were 2-tailed, and a p value<0.05 was considered statistically significant. SAS 9.1 software (SAS Institute Inc., Cary, NC, USA) was used for all analyses.

## Results

### Standard incidence of overall arrest

**Fig 1:**
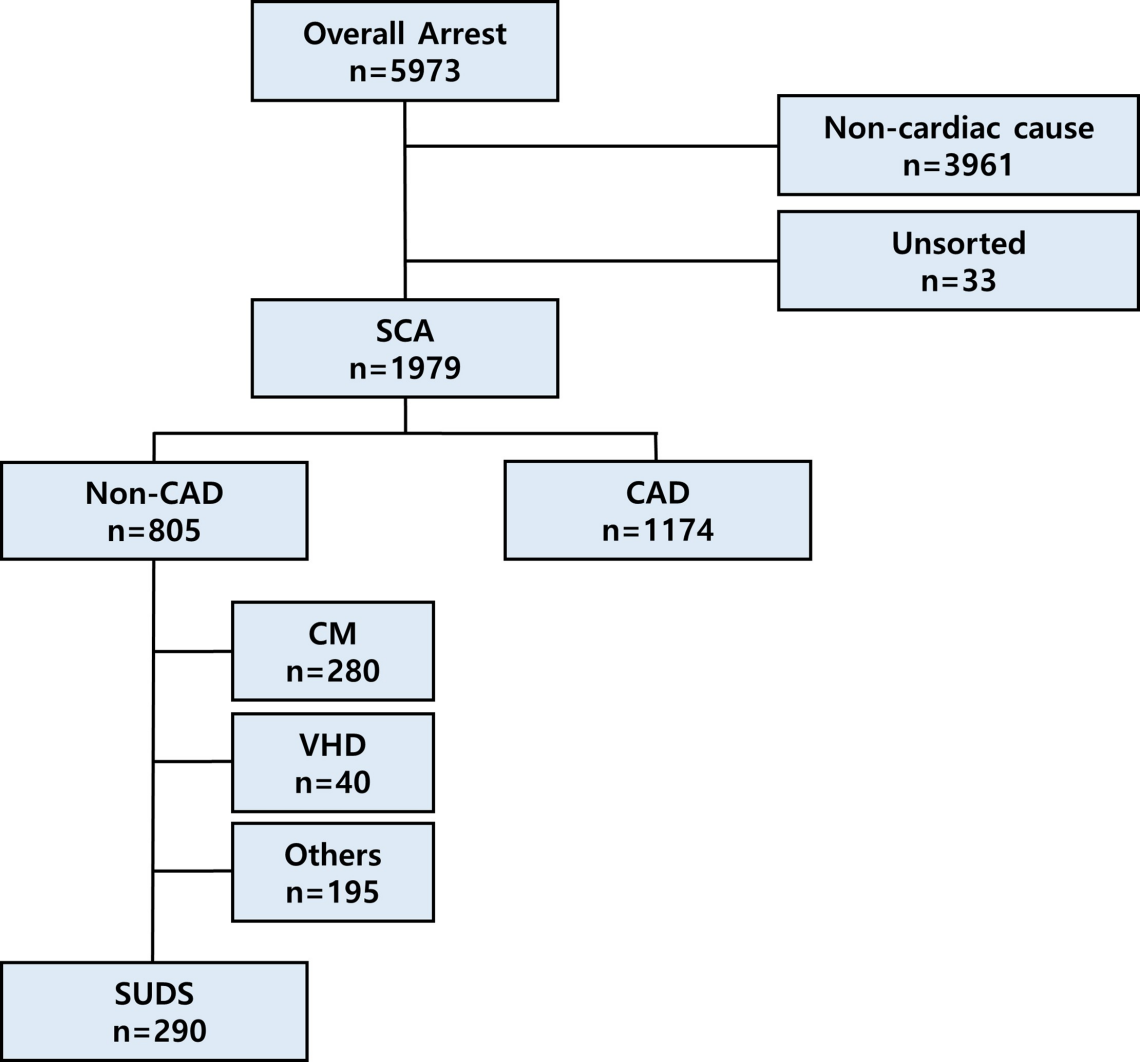
Schematic representation of study population selection and sudden cardiac arrest case adjudication as etiology. SCA, sudden cardiac arrest; CAD, coronary artery disease; CM, cardiomyopathy; VHD, valvular heart disease; SUDS, sudden unexplained death syndrome.

Between Jan 1, 2002 and Dec 31, 2013, a total of 1,125,691 subjects were monitored in this prospective cohort observational study. Over the 12-year study period, 5973 patients from the cohort (0.53% of total cohort) suffered arrest (Fig 1). Of these, 3452 (57.8%) were male and 2521 (42.2%) were female. The incidence of overall arrest was 48.7 per 100,000 person-years (95% CI 47.5–50.0). The incidence of overall arrest was higher in male than in female (56.3 per 100,000 person-years [95% CI 54.4–58.2] and 41.2 per 100,000 person-years [95% CI 39.6–42.8] for male and female, respectively).

### Incidence and demographic findings of patients with sudden cardiac arrest

**Fig 2:**
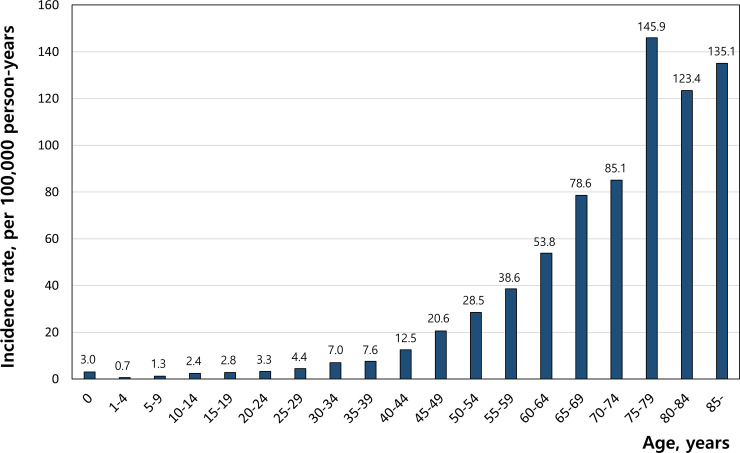
Annual age-related distribution of sudden cardiac arrest incidence per 100,000 person-years.

**Fig 3:**
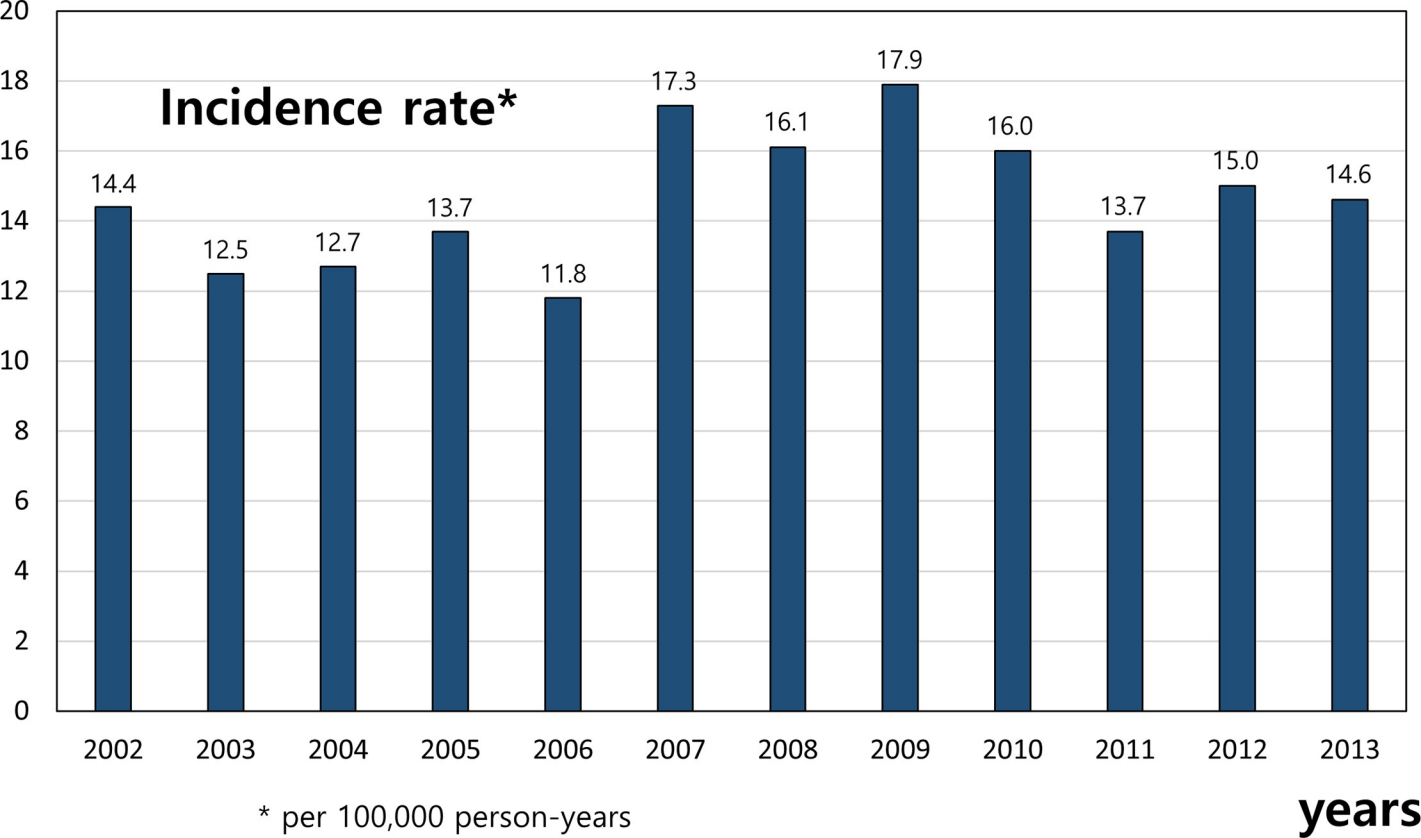
Trends in age- and sex-adjusted annual incidence of sudden cardiac arrest per 100,000 person-years.

After disease code review, secondary cardiac arrest due to non-cardiac cause was excluded to estimate the primary SCA. There were 1979 primary SCA cases (0.17% of total cohort). The incidence of SCA was 16.1 per 100,000 person-years (95% CI 15.51–49.98). The incidence of SCA was 18.1 per 100,000 person-years in male (95% CI 17.1–19.2) and 14.1 per 100,000 person-years in female (95% CI 13.2–15.1). Fig 2 shows the significant difference in incidence of SCA by age. The incidence was 3.01 per 100,000 person-years among subjects less than one year of age and decreased from one to four years of age, but then increased continuously with age. Most cases occurred in subjects 65 years and older, with the peak between the ages of 75 to 79. Fig 3 shows the annual incidence of SCA during the 12-year cohort period. The rate has increased since 2007. Table 1 shows the baseline characteristics of these groups. There were 1,147 (59.3%) patients with CAD and 805 (40.7%) patients did not have CAD. More than half of patients were male (56.3%) in the total SCA group. The proportion of males was higher in the CAD group (60.3% vs. 50.4%, p < 0.001). The percentages of patients with hypertension, diabetes mellitus, chronic kidney disease, cerebrovascular accident and malignancy were significantly higher in the CAD group than in the non-CAD group (p<0.05 for all). Table 2 showed the risk factor associated with SCA. The risk of SCA was significantly reduced until the age of 9 and then was significantly higher at the age of over 30. Incidences in teens or twenties were lower than in the reference age. Men had 1.647 times of the SCA risk compared to women (C.I 1.503–1.804, p<0.001). Among the risk factors, heart failure had highest risk for SCA (OR = 3.667, C.I 3.284–4.095, p<0.0001). Hypertension, diabetes, coronary artery disease, heart failure, chronic kidney disease, cerebrovascular accident and atrial fibrillation were related with high risk of SCA (all p values <0.0001), but chronic lung disease was not. Reversely, people with malignancy had a lower risk of SCA than those without one.

**Table 1:** Clinical characteristics of the patient with sudden cardiac arrest.

	Total SCA n = 1979	CAD n = 1174	Non-CAD n = 805	p value
Sex, males, n (%)	1114 (56.3)	708 (60.3)	406 (50.4)	<0.001
Hypertension, n (%)	1536 (77.6)	985 (83.9)	551 (68.4)	<0.001
Diabetes mellitus, n (%)	571 (28.9)	386 (32.9)	185 (23.0)	<0.001
Heart failure, n (%)	755 (38.2)	459 (39.1)	296 (36.8)	0.150
Chronic kidney disease, n (%)	122 (6.2)	83 (7.1)	39 (4.8)	0.046
Chronic lung disease, n (%)	70 (3.5)	45 (3.8)	25 (3.1)	0.229
Cerebrovascular accident, n (%)	608 (30.7)	403 (34.3)	205 (25.5)	<0.001
Atrial fibrillation, n (%)	421 (21.3)	248 (21.1)	173 (21.5)	0.453
Malignancy, n (%)	188 (9.5)	125 (10.6)	63 (7.8)	0.035

Values are given as number (percent)

SCA, sudden cardiac arrest; CAD, coronary artery disease.

**Table 2:** Multivariate analysis of factors associated with sudden cardiac arrest.

Risk factor		Odds ratio (95% CI)	p Value
**Age**	**0 (Reference)**	1	
	**1–4**	0.216 (0.074–0.628)	0.005
	**5–9**	0.409 (0.197–0.848)	0.016
	**10–14**	0.767 (0.416–1.415)	0.395
	**15–19**	0.874 (0.484–1.579)	0.655
	**20–24**	0.985 (0.568–1.708)	0.956
	**25–29**	1.247 (0.741–2.098)	0.406
	**30–34**	1.824 (1.126–2.954)	0.015
	**35–39**	1.743 (1.073–2.831)	0.025
	**40–44**	2.422 (1.520–3.861)	0.000
	**45–49**	3.283 (2.067–5.217)	< .0001
	**50–54**	3.804 (2.388–6.059)	< .0001
	**55–59**	4.200 (2.634–6.695)	< .0001
	**60–64**	4.787 (3.013–7.606)	< .0001
	**65–69**	6.022 (3.787–9.577)	< .0001
	**70–74**	5.870 (3.659–9.417)	< .0001
	**75–79**	9.931 (6.184–15.948)	< .0001
	**80–84**	9.016 (5.443–14.933)	< .0001
	**85-**	13.243 (7.621–23.012)	< .0001
**Male sex (vs Female)**		1.645 (1.501–1.802)	< .0001
**Hypertension**		2.270 (1.979–2.604)	< .0001
**Diabetes mellitus**		1.231 (1.109–1.366)	< .0001
**Coronary artery disease**		1.559 (1.349–1.802)	< .0001
**Heart failure**		3.551 (3.177–3.968)	< .0001
**Chronic kidney disease**		1.502 (1.245–1.812)	< .0001
**Chronic lung disease**		0.841 (0.660–1.072)	0.163
**Cerebrovascular accident**		1.244 (1.118–1.384)	< .0001
**Atrial fibrillation**		3.133 (2.774–3.538)	< .0001
**Malignancy**		0.773 (0.663–0.902)	0.001

CI, confidence interval

**Fig 4:**
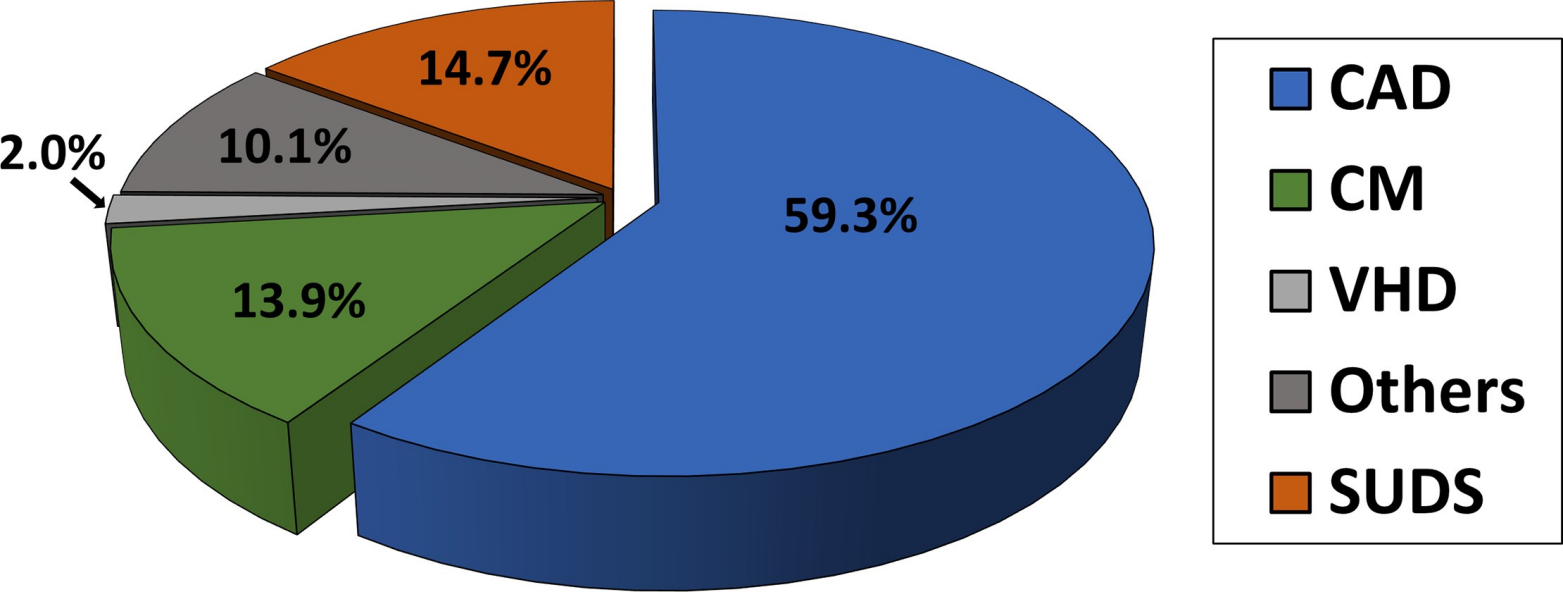
Causes of sudden cardiac arrest. Coronary artery disease was responsible for 59.3% of cases, followed by cardiomyopathy (13.9%), and valvular heart disease (2%). Sudden unexplained death syndrome was 14.7% of total sudden cardiac arrest, CAD. coronary artery disease; CM. cardiomyopathy; VHD. valvular heart disease; SUDS. sudden unexplained death syndrome.

**Fig 5:**
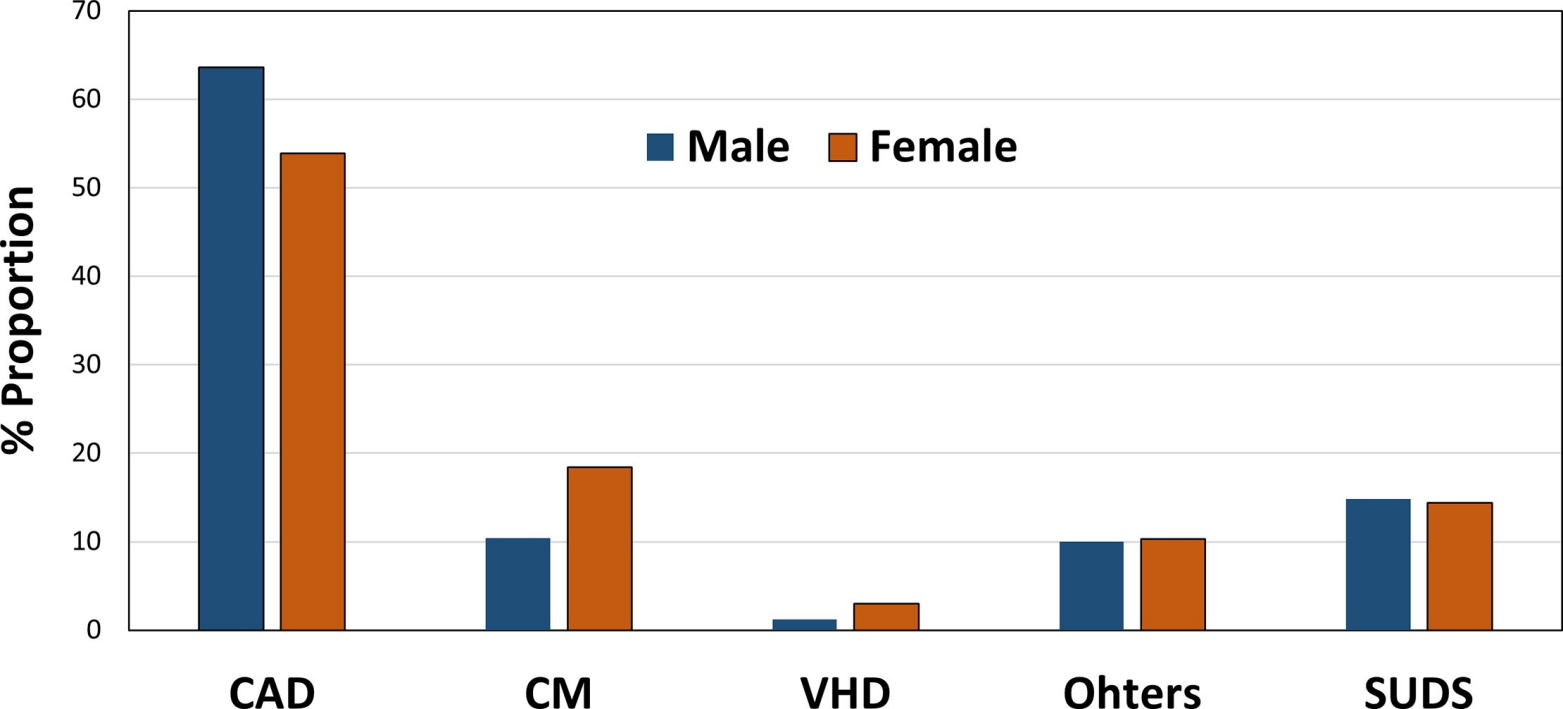
Sex-based distribution of presumed etiologies of sudden cardiac arrest. CAD, coronary artery disease; CM, cardiomyopathy; VHD, valvular heart disease; SUDS, sudden unexplained death syndrome.

CAD (n = 1174) was the most common cause, accounting for 59.3% of SCA cases (Fig 4). The second most common cause was CM (13.9%) and VHD followed (2.0%). SUDS accounts for 14.7% of total SCA cases. Fig 5 shows the sex-based distribution of presumed SCA etiologies. CAD was the most common cause of SCA in both sexes (63.6% in males vs. 53.9% in females, p < 0.0001). SCA related to CM (10.4% in males vs. 18.4 in females, p < 0.0001) and VHD (1.2% in males vs. 3% in females, p = 0.0061) were significantly higher in female. The proportion of SUDS was similar for males and females (14.8% in males vs. 14.4% in females, p = 0.822).

### Estimated incidence of sudden unexplained death syndrome

**Fig 6:**
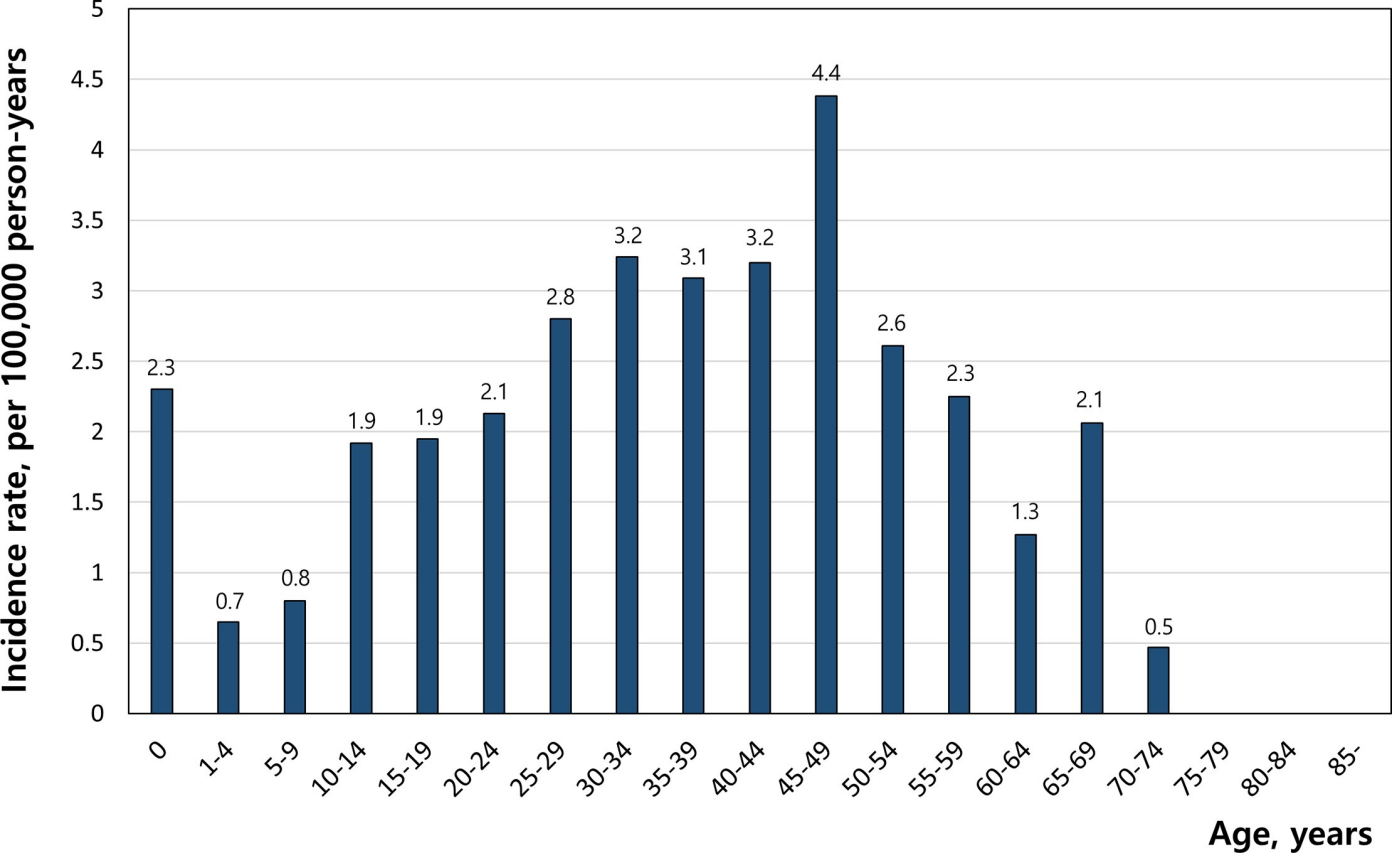
Annual age-related distribution of sudden unexplained death syndrome incidence per 100,000 person-years.

The total number of estimated SUDS was 290, this was 0.026% of the total cohort. Table 3 shows the baseline characteristics of these patients. Among them, 165 (56.9%) were male and 125 (43.1%) were female. The incidence of SUDS was 2.36 per 100,000 person-years (95% CI 2.11–2.65). It was 2.69 per 100,000 person-years (95% CI 2.31–3.13) in male and 2.04 in female (95% CI 1.71–2.43). There was no difference in incidence by year (S1 Fig). The 30- to 50-year–old interval showed the highest incidence of SCA (Fig 6). The highest incidence was 4.38 per 100,000 person-years for those aged 45–49 years.

**Table 3:** Clinical characteristics of the patient with sudden unexplained death syndrome.

	Total sudden unexplained death syndrome n = 290
**Sex, males, n (%)**	165 (56.9)
**Hypertension, n (%)**	90 (31.)
**Diabetes mellitus, n (%)**	21 (7.2)
**Heart failure, n (%)**	6 (2.1)
**Chronic kidney disease, n (%)**	1 (0.3)
**Chronic lung disease, n(%)**	2 (0.7)
**Cerebrovascular accident, n (%)**	15 (5.2)
**Atrial fibrillation, n (%)**	21 (7.2)
**Malignancy, n (%)**	6 (2.1)

Values are given as number (percent)

### Regional and seasonal differences in incidence of sudden cardiac arrest and sudden unexplained death syndrome

The incidence of SCA and SUDS show regional differences (S1 Table). The regional analysis showed no significant difference in incidence between seven metropolitan areas including Seoul and rural areas. The areas with the highest SCA incidence per 100,000 person-years were Jeollabuk-do (24.4), Gangwon-do (24.0), and Busan (22.2) (S2 Fig). The area with the lowest incidence was Daegu (7.9). The areas with the highest incidence of SUDS were Jeollabuk-do (4.8), Gwangju (3.9), and Ulsan (3.18), and the area with the lowest was Daegu (1.1) (S3 Fig). There was a lower incidence of SCA in autumn than in spring, summer, and winter (S4 Fig). The hazard ratio was 1.13 (0.925–1.381) in spring, 0.93 (95% CI 0.755–1.145) in summer, and 0.561 (95% CI 0.457–0.69) in autumn compared to winter. The incidence of SUDS in autumn (0.118 [95% CI 0.049–0.282]) was lower than that of winter (S5 Fig).

## Discussion

This study is the first nationwide, community-based investigation assessing the incidence rate of SCA and SUDS in Korea. We examined 5973 overall arrests and 1979 primary SCA subjects who were extracted from a national database of 1,125,691 randomly selected subjects. We found that SUDS accounts for 14.7% of primary SCA in Korea.

In other studies based on data from Western countries, the incidence of SCA was found to be between 50 and 100 per 100,000 person-years [10, 11]. Recent prospective studies showed an incidence of 52–53 per 100,000 person-years in North America, and 51.2 in Ireland [2, 12, 13]. In Asia, lower incidence rates for SCA are reported than in Western countries. Wei et al. report an incidence of 41.8 per 100,000 person-years in China [14]. In this study, population in the four representative cities were selected and the death certification or ICD code was used for SCA diagnosis. CAD was 60% of SCA etiology in China. Maruyama et al. showed 36.8 per 100,000 person-years in Japan [6]. ICD code and chart review were used for SCA diagnosis. SCA related myocardial infarction is less than 40% in Japan. In our study, the incidence of overall arrests was 47.8 per 100,000 person-years in Korea. The Korean incidence is lower than in the West and higher than that seen in other East Asian countries. CAD was 60% of SCA etiology. The proportion of CAD in Korea was similar to China and was lowest in Japan. Other etiology was not evaluated in Chinese and Japanese’s studies. In Korea, the out-of-hospital cardiac arrest (OHCA) rate was surveyed based on emergency medical services’ (EMS) data. Ro et al. found that the mean incidence of OHCAs was 42.6 per 100,000 person-years (37.5 in 2006; 39.8 in 2007; 42.5 in 2008; 45.6 in 2009; and 46.8 in 2010 per 100,000 person-years) in Korea [15]. Ahn et al. reported that the annual incidence of non-traumatic OHCA was 20.9 (2006) and 22.2 (2007) per 100,000 people in Korea [16]. The incidence of SCA reported in several studies has varied depending on definition and method. We defined SCA broadly as overall arrests to reduce missed cases. The criteria used to define primary cardiac arrest were strict, and people with possible non-cardiac origin for SCA were excluded. As a result, there was a higher incidence of overall arrest than reported in other Korean studies, and there was a relatively low incidence of primary cardiac arrest. The cohort in Ahn et al. included both cardiac and respiratory arrest cases. The incidence of pure cardiac arrest was estimated to be 16–20 per 100,000 person-years in Korea, based on several studies including ours. While SCA has been on a declining trend worldwide, recent Asian data shows an increased incidence since 2000. Maruyama’s data showed that the incidence per 100,000 person-years was 76.0 in 1981–1985, 57.9 in 1986–1990, 39.3 in 1991–1995, 31.6 in 1996–2000, and 36.8 in 2001–2005. Korean data including ours have shown an increase in incidence since 2000 [6]. This suggests that other resolutions beyond traditional CAD-focused SCA prevention are needed.

CAD is the most common substrate underlying SCA in Western countries, responsible for 75% of SCAs [10, 17, 18]. These results are different in Asians. According to Japanese and Chinese data, CAD caused less than half of the SCAs in Asian males and females. This led a relatively low prevalence of SCA compared to Western countries. The prevalence of SCA in Korea was also lower than that in Western countries. However, the proportion of CAD as the cause of SCA was not reported in this study. As with other studies in Asia, our data showed a lower incidence of SCA compared to that of Western countries, but the proportion of CAD was greater than 50% because our definition of CAD included everyone with a history of myocardial ischemia and related procedure codes. Clinical data often were not enough to determine an exact main cause because of the nature of SCA. In 5% of SCAs, a significant cardiac abnormality is not found after extensive evaluation or at autopsy [19, 20]. Therefore, we collected retrospective patient histories of CVD to explore possible associations with SCA. Study design also can lead to differences in results.

Seasonal and regional data provide a clue as to the cause of the SCA. The seasonal differences in SUDS incidence can be attributed to the effects of temperature. Some Western data show that SCA occurs more frequently during the cold season [21] or spring [22]. It is usually related to seasonal variations in coronary artery disease occurrence. The results of our present study differ somewhat from previous results, and this difference may be due to geographical and climatic variations. SUDS showed a similar seasonal pattern to SCA. In the case of Brugada syndrome or J wave syndrome, changes in the electrocardiogram or SCA were related to body temperature [23, 24]. Brugada syndrome can also be unmasked by prevalent viral infections and drug use. The regional differences are more complex and may relate to differences in air pollution, accessibility of hospitals, and population distribution. South Korea is mainly single ethnic group, and the racial background of Korea is very uniform. Jeollabuk-do, Gangwon-do and Gyeongsangnam-do with the relatively high incidence rates of SCA are rural areas. We speculate that accessibility to hospital and relatively older population may impact on the result. However, we think that the exact cause of regional difference for incidences of SUDS is unknown. This finding would be studied focusing on whether there is different in racial distribution among regions and the genetic heterogeneity in the future. Detailed data analysis is needed to identify prevention methods.

We explored the proportion of SUDS using nation-wide population data. It was estimated to be 14.7% of all SCA cases. The incidence of SUDS was 2.36 per 100,000 person-years. In previous studies, the incidence of SUDS among the young and middle-aged general population was estimated to be 0.76 to 1.34/100,000 annually [8, 25] and SUDS accounted for 4–24% of the SCA cases [9, 25]. It was estimated to be related to 10% of SCA events in Asia [26, 27]. The difference in incidence is due to the inclusion of elderly patients who were excluded from previous studies. Elderly patients without a specific code or past history were classified as SUDS even if they died from CAD or structural HD. The difference in the study population was the reason for the higher incidence of SUDS compared to other studies. Among large number of SCA cases, autopsy was not performed. The total autopsy rate is known to 2~3% of SCA in South Korea. It is lower compared to that of the western countries. It can also influence the high rate of SUDS. In the previous Korean data about OHCA from a single center, 44% of SCA were SUDS without structural HD [28]. SCAs due to ICA are included in SUDS in this study. ICAs causing SCA were long or short QT syndrome, Brugada syndrome, catecholaminergic polymorphic ventricular tachycardia, and early repolarization syndrome. In our study, it was difficult to identify the specific diagnosis of ICA because disease codes did not exist in the Korean Standard Classification of Diseases (KCD)-4,5,6 used until 2016. However, in KCD-7, codes for each ICA were newly established, and classification will be possible later.

This was a cohort study based on real-world registry data. This study has several limitations that should be considered when interpreting the results. A major limitation is the possibility of misclassification of SCA cases. For those who died out-of-hospital, the cause of death was estimated based on the death certificate and previous medical records. We used disease and procedure codes, which it could have been impacted by the clinician’s judgement. There is a possibility that some SCAs could be misclassified as non-SCA, which could underestimate SCA in this population. Another limitation is the possibility that we overestimated the SUDS rate among SCA cases. Some false-positive misclassifications may have occurred because post-mortem autopsies are rarely performed. Some elderly patients with SCA who did not undergo autopsy can be classified with SUDS. SUDS accounts for about 10–15% of SCA considering this overestimation.

## Conclusions

We analyzed the epidemiologic data for SCA and SUDS in the Korean population. The standardized incidence of overall sudden arrest and SCA in Koreans was 48.7 and 16.1 per 100,000 person-years, respectively. SUDS accounted for 14.7% of total SCAs, a significant proportion. The proportion of SUDS among SCA were higher than previously reported.

## Supporting information

S1 Fig Trends in age- and sex-adjusted annual incidence of sudden unexplained death syndrome per 100,000 person-years. (TIF)

S2 Fig Regional distribution of sudden cardiac arrest incidence per 100,000 person-years in Korea. (TIF)

S3 Fig Regional distribution of sudden cardiac arrest from inherited cardiac channelopathies incidence per 100,000 person-years in Korea. (TIF)

S4 Fig Hazard ratio (HR) and 95% confidence interval (CI) for sudden cardiac arrest by season, adjusted for age, with winter as the reference category. (TIF)

S5 Fig Hazard ratio (HR) and 95% confidence interval (CI) for sudden unexplained death syndrome by season, adjusted for age, with winter as the reference category. (TIF)

S1 Table Region related distribution of sudden cardiac arrest and sudden unexplained death syndrome incidence rate in Korea. (DOCX)

## References

[1] Eckart RE, Shry EA, Burke AP, McNear JA, Appel DA, Castillo-Rojas LM, et al Sudden death in young adults: an autopsy-based series of a population undergoing active surveillance. J Am Coll Cardiol. 2011; 58:1254–1261. 10.1016/j.jacc.2011.01.049 21903060

[2] Chugh SS, Jui J, Gunson K, Stecker EC, John BT, Thompson B, et al Current burden of sudden cardiac death: multiple source surveillance versus retrospective death certificate-based review in a large U.S. community. J Am Coll Cardiol. 2004; 44:1268–1275. 10.1016/j.jacc.2004.06.029 15364331

[3] Adabag AS, Luepker RV, Roger VL, Gersh BJ. Sudden cardiac death: epidemiology and risk factors. Nat Rev Cardiol. 2010; 7:216–225. 10.1038/nrcardio.2010.3 20142817 PMC5014372

[4] Ackerman MJ, Priori SG, Willems S, Berul C, Brugada R, Calkins H, et al HRS/EHRA expert consensus statement on the state of genetic testing for the channelopathies and cardiomyopathies this document was developed as a partnership between the Heart Rhythm Society (HRS) and the European Heart Rhythm Association (EHRA). Heart Rhythm. 2011; 8:1308–1339. 10.1016/j.hrthm.2011.05.020 21787999

[5] Myerburg RJ. Sudden cardiac death: exploring the limits of our knowledge. J Cardiovasc Electrophysiol. 2001; 12:369–381. 10.1046/j.1540-8167.2001.00369.x 11291815

[6] Maruyama M, Ohira T, Imano H, Kitamura A, Kiyama M, Okada T, et al Trends in sudden cardiac death and its risk factors in Japan from 1981 to 2005: the Circulatory Risk in Communities Study (CIRCS). BMJ Open. 2012; 2:e000573 10.1136/bmjopen-2011-000573 22446988 PMC3312077

[7] Behr E, Wood DA, Wright M, Syrris P, Sheppard MN, Casey A, et al Cardiological assessment of first-degree relatives in sudden arrhythmic death syndrome. Lancet. 2003; 362:1457–1459. 10.1016/s0140-6736(03)14692-2 14602442

[8] Behr ER, Casey A, Sheppard M, Wright M, Bowker TJ, Davies MJ, et al Sudden arrhythmic death syndrome: a national survey of sudden unexplained cardiac death. Heart. 2007; 93:601–605. 10.1136/hrt.2006.099598 17237131 PMC1955564

[9] Bowker TJ, Wood DA, Davies MJ, Sheppard MN, Cary NR, Burton JD, et al Sudden, unexpected cardiac or unexplained death in England: a national survey. QJM. 2003; 96:269–279. 10.1093/qjmed/hcg038 12651971

[10] de Vreede-Swagemakers JJ, Gorgels AP, Dubois-Arbouw WI, van Ree JW, Daemen MJ, Houben LG, et al Out-of-hospital cardiac arrest in the 1990's: a population-based study in the Maastricht area on incidence, characteristics and survival. J Am Coll Cardiol. 1997; 30:1500–1505. 10.1016/s0735-1097(97)00355-0 9362408

[11] Vaillancourt C, Stiell IG, Canadian Cardiovascular Outcomes Research T. Cardiac arrest care and emergency medical services in Canada. Can J Cardiol. 2004; 20:1081–1090. 15457303

[12] Nichol G, Thomas E, Callaway CW, Hedges J, Powell JL, Aufderheide TP, et al Regional variation in out-of-hospital cardiac arrest incidence and outcome. JAMA. 2008; 300:1423–1431. 10.1001/jama.300.12.1423 18812533 PMC3187919

[13] Byrne R, Constant O, Smyth Y, Callagy G, Nash P, Daly K, et al Multiple source surveillance incidence and aetiology of out-of-hospital sudden cardiac death in a rural population in the West of Ireland. Eur Heart J. 2008; 29:1418–1423. 10.1093/eurheartj/ehn155 18424446

[14] Hua W, Zhang LF, Wu YF, Liu XQ, Guo DS, Zhou HL, et al Incidence of sudden cardiac death in China: analysis of 4 regional populations. J Am Coll Cardiol. 2009; 54:1110–1118. 10.1016/j.jacc.2009.06.016 19744622

[15] Ro YS, Shin SD, Song KJ, Lee EJ, Kim JY, Ahn KO, et al A trend in epidemiology and outcomes of out-of-hospital cardiac arrest by urbanization level: a nationwide observational study from 2006 to 2010 in South Korea. Resuscitation. 2013; 84:547–557. 10.1016/j.resuscitation.2012.12.020 23313428

[16] Ahn KO, Shin SD, Suh GJ, Cha WC, Song KJ, Kim SJ, et al Epidemiology and outcomes from non-traumatic out-of-hospital cardiac arrest in Korea: A nationwide observational study. Resuscitation. 2010; 81:974–981. 10.1016/j.resuscitation.2010.02.029 20605312

[17] Spain DM, Bradess VA, Mohr C. Coronary atherosclerosis as a cause of unexpected and unexplained death. An autopsy study from 1949–1959. JAMA. 1960; 174:384–388. 10.1001/jama.1960.03030040038010 13833138

[18] Manfredini R, Portaluppi F, Grandi E, Fersini C, Gallerani M. Out-of-hospital sudden death referring to an emergency department. J Clin Epidemiol. 1996; 49:865–868. 10.1016/0895-4356(96)00114-x 8699205

[19] Priori SG, Borggrefe M, Camm AJ, Hauer RN, Klein H, Kuck KH, et al Unexplained cardiac arrest. The need for a prospective registry. Eur Heart J. 1992; 13:1445–1446. 10.1093/oxfordjournals.eurheartj.a060083 1464332

[20] Chugh SS, Kelly KL, Titus JL. Sudden cardiac death with apparently normal heart. Circulation. 2000; 102:649–654. 10.1161/01.cir.102.6.649 10931805

[21] Arntz HR, Willich SN, Schreiber C, Bruggemann T, Stern R, Schultheiss HP. Diurnal, weekly and seasonal variation of sudden death. Population-based analysis of 24,061 consecutive cases. Eur Heart J. 2000; 21:315–320. 10.1053/euhj.1999.1739 10653679

[22] Kriszbacher I, Boncz I, Koppan M, Bodis J. Seasonal variations in the occurrence of acute myocardial infarction in Hungary between 2000 and 2004. Int J Cardiol. 2008; 129:251–254. 10.1016/j.ijcard.2007.07.095 18023894

[23] Stroker E, de Asmundis C, Chierchia GB, Brugada P. Exercise-related Brugada pattern and monomorphic ventricular tachycardia in a patient with Brugada syndrome: interplay between body temperature, haemodynamics and vagal activity. Eur Heart J. 2016; 37:655 10.1093/eurheartj/ehv263 26074464

[24] Maeda S, Takahashi Y, Nogami A, Yamauchi Y, Osaka Y, Shirai Y, et al Seasonal, weekly, and circadian distribution of ventricular fibrillation in patients with J-wave syndrome from the J-PREVENT registry. J Arrhythm. 2015; 31:268–273. 10.1016/j.joa.2015.01.004 26550081 PMC4600836

[25] Margey R, Roy A, Tobin S, O'Keane CJ, McGorrian C, Morris V, et al Sudden cardiac death in 14- to 35-year olds in Ireland from 2005 to 2007: a retrospective registry. Europace. 2011; 13:1411–1418. 10.1093/europace/eur161 21798877

[26] Murakoshi N, Aonuma K. Epidemiology of arrhythmias and sudden cardiac death in Asia. Circ J. 2013; 77:2419–2431. 10.1253/circj.cj-13-1129 24067274

[27] Roh SY, Choi JI, Kim MS, Cho EY, Kim YG, Lee KN, et al Trends in the use of implantable cardioverter-defibrillators for prevention of sudden cardiac arrest: A South Korean nationwide population-based study. Pacing Clin Electrophysiol. 2019; 42:1086–1094. 10.1111/pace.13741 31197835

[28] Cho JG, Park HW, Rhew JY, Lee SR, Chung WK, Park OY, et al Clinical characteristics of unexplained sudden cardiac death in Korea. Jpn Circ J. 2001; 65:18–22. 10.1253/jcj.65.18 11153816

