# Supplementary figures and images for: Incidence and etiology of sudden cardiac arrest in Koreans: A cohort from the national health insurance service database
Source: PLoS One. 2020 Nov 25;15(11):e0242799. doi: 10.1371/journal.pone.0242799 (PMC7688167; doi:10.1371/journal.pone.0242799)

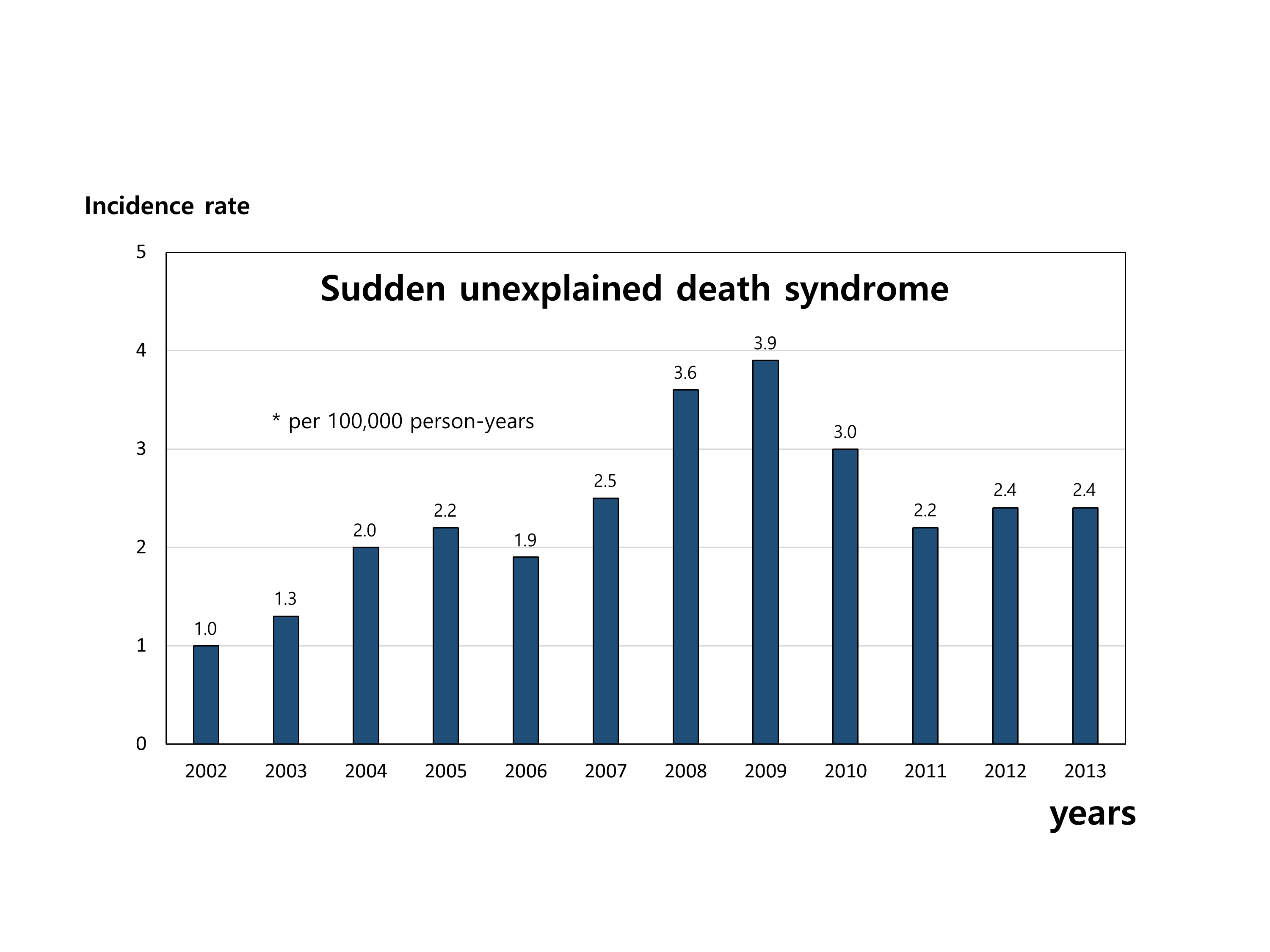

Supplement: S1 Fig — (TIF) [file pone.0242799.s001.TIF]

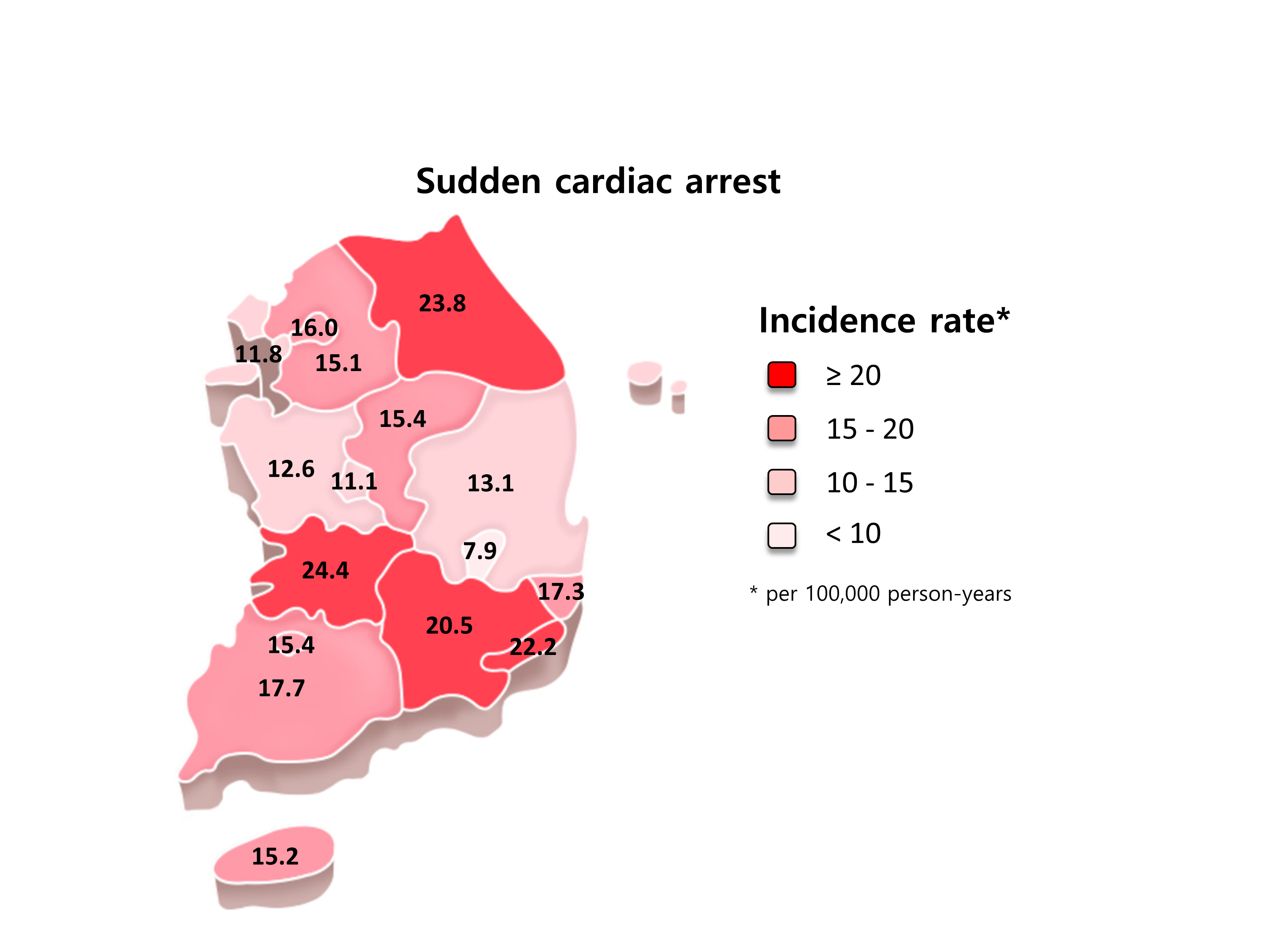

Supplement: S2 Fig — (TIF) [file pone.0242799.s002.TIF]

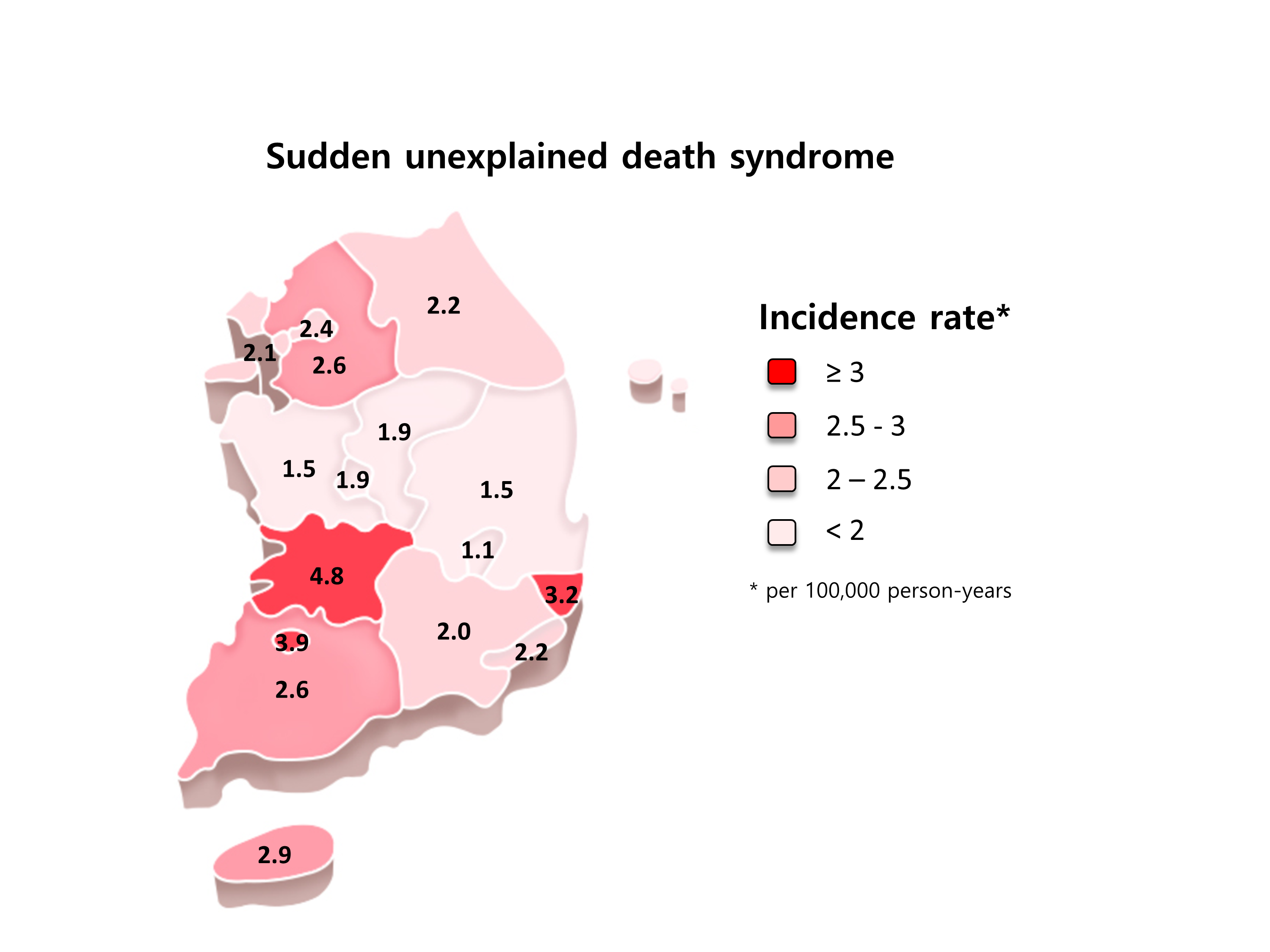

Supplement: S3 Fig — (TIF) [file pone.0242799.s003.TIF]

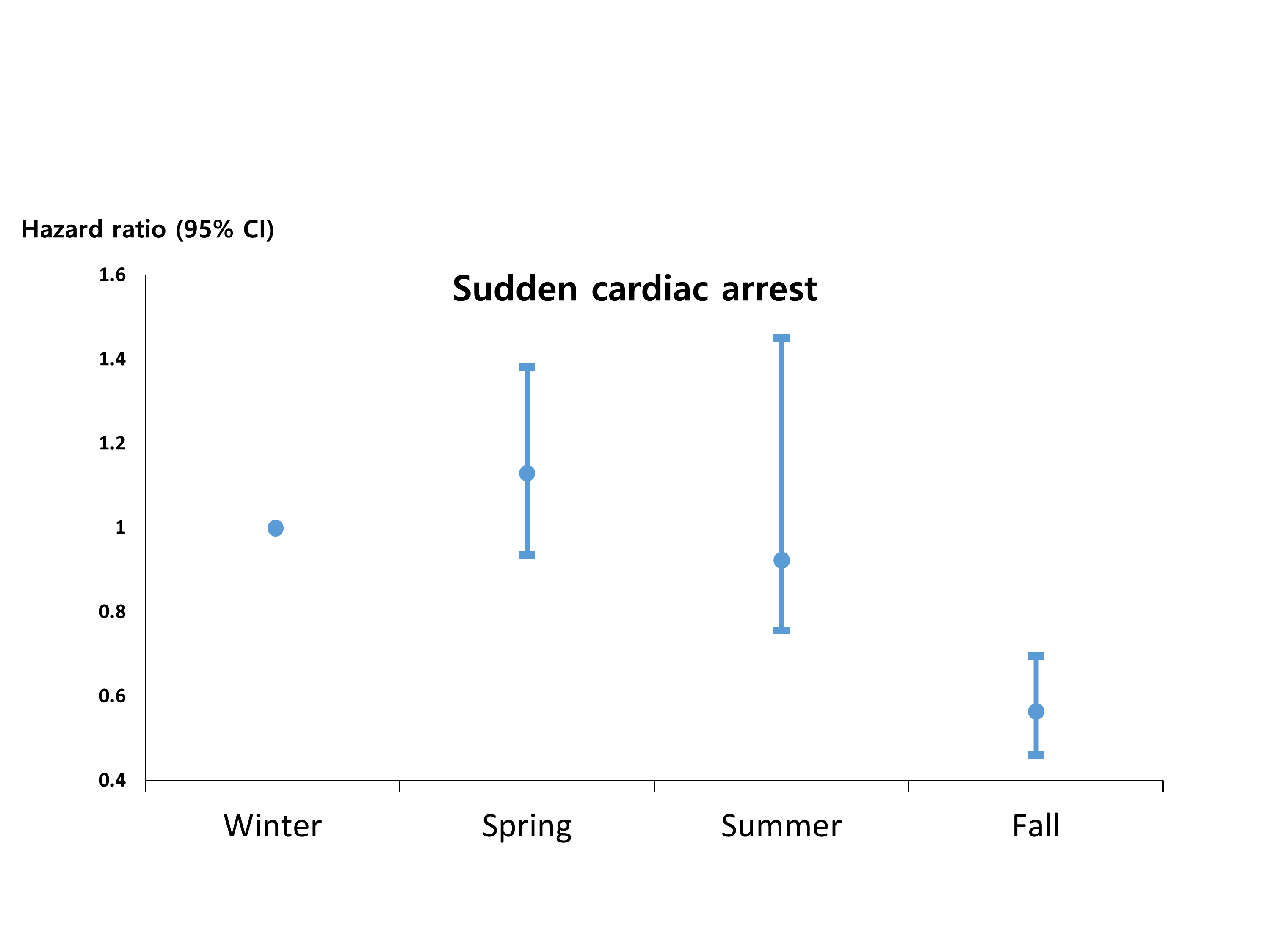

Supplement: S4 Fig — (TIF) [file pone.0242799.s004.TIF]

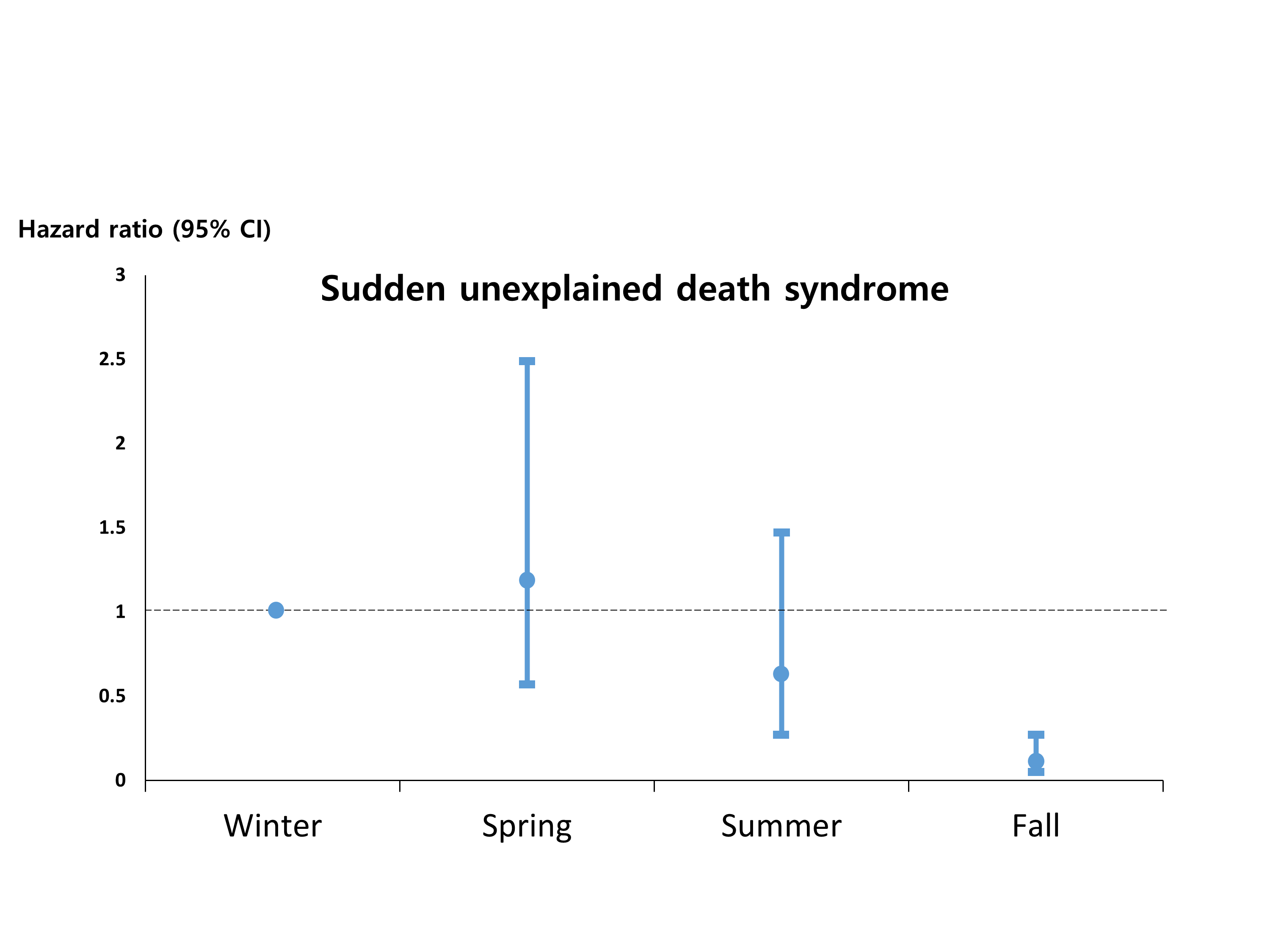

Supplement: S5 Fig — (TIF) [file pone.0242799.s005.TIF]
