# Supplementary material for: Incidence and etiology of sudden cardiac arrest in Koreans: A cohort from the national health insurance service database
Source: PLoS One. 2020 Nov 25;15(11):e0242799. doi: 10.1371/journal.pone.0242799 (PMC7688167; doi:10.1371/journal.pone.0242799)
Supplement: S1 Table — (DOCX) [file pone.0242799.s006.docx]

**S1 Table. Region related distribution of sudden cardiac arrest and sudden unexplained death syndrome incidence rate in Korea.**

| Region | Total cohort | SCA | | SUDS | |
| --- | --- | --- | --- | --- | --- |
|  |  | **N** | **Incidence rate^a^** | **N** | **Incidence rate^b^** |
| Republic of Korea | 1125691 | 1979 | 16.1 | 290 | 2.4 |
| Seoul | 237638 | 418 | 16.0 | 63 | 2.4 |
| Busan | 85293 | 210 | 22.2 | 21 | 2.2 |
| Daegu | 58225 | 51 | 7.9 | 7 | 1.1 |
| Incheon | 60029 | 78 | 11.8 | 14 | 2.1 |
| Gwangju | 33141 | 56 | 15.4 | 14 | 3.9 |
| Daejeon | 33651 | 41 | 11.1 | 7 | 1.9 |
| Ulsan | 25613 | 49 | 17.3 | 9 | 3.2 |
| Gyeonggi-do | 237118 | 388 | 15.1 | 67 | 2.6 |
| Gangwon-do | 34397 | 88 | 23.8 | 8 | 2.2 |
| Chungcheongbuk-do | 34806 | 58 | 15.4 | 7 | 1.9 |
| Chungcheongnam-do | 44900 | 61 | 12.6 | 7 | 1.5 |
| Jeollabuk-do | 44740 | 118 | 24.4 | 23 | 4.8 |
| Jeollanam-do | 47060 | 90 | 17.7 | 13 | 2.6 |
| Gyeongsangbuk-do | 63324 | 90 | 13.1 | 10 | 1.5 |
| Gyeongsangnam-do | 72773 | 162 | 20.5 | 16 | 2.0 |
| Jeju | 12918 | 21 | 15.2 | 4 | 2.9 |

^a^ Incidence rate of SCA (per 100,000 person-years), ^b^ Incidence rate of SUDS (per 100,000 person-years)

N, number; SCA, sudden cardiac arrest; SUDS, sudden unexplained death syndrome.
